# Influence of Spatial Resolution in Three-dimensional Cine Phase Contrast Magnetic Resonance Imaging on the Accuracy of Hemodynamic Analysis

**DOI:** 10.2463/mrms.mp.2016-0060

**Published:** 2017-01-30

**Authors:** Atsushi Fukuyama, Haruo Isoda, Kento Morita, Marika Mori, Tomoya Watanabe, Kenta Ishiguro, Yoshiaki Komori, Takafumi Kosugi

**Affiliations:** 1 Department of Radiological and Medical Laboratory Sciences, Nagoya University Graduate School of Medicine, 1-1-20 Daiko-Minami, Higashi-ku, Nagoya, Aichi 461-8673, Japan; 2 Brain & Mind Research Center, Nagoya University, Aichi, Japan; 3 Toyohashi Municipal Hospital, Aichi, Japan; 4 Gifu Prefectural General Medical Center, Gifu, Japan; 5 Kyoto University Hospital, Kyoto, Japan; 6 Siemens Healthcare K.K. Tokyo, Japan; 7 Renaissance of Technology Corporation, Shizuoka, Japan

**Keywords:** three dimensional cine phase contrast magnetic resonance imaging, hemodynamics, spatial resolution, spatially-averaged velocity, maximum velocity

## Abstract

**Introduction:**

We aim to elucidate the effect of spatial resolution of three-dimensional cine phase contrast magnetic resonance (3D cine PC MR) imaging on the accuracy of the blood flow analysis, and examine the optimal setting for spatial resolution using flow phantoms.

**Materials and Methods:**

The flow phantom has five types of acrylic pipes that represent human blood vessels (inner diameters: 15, 12, 9, 6, and 3 mm). The pipes were fixed with 1% agarose containing 0.025 mol/L gadolinium contrast agent. A blood-mimicking fluid with human blood property values was circulated through the pipes at a steady flow. Magnetic resonance (MR) images (three-directional phase images with speed information and magnitude images for information of shape) were acquired using the 3-Tesla MR system and receiving coil. Temporal changes in spatially-averaged velocity and maximum velocity were calculated using hemodynamic analysis software. We calculated the error rates of the flow velocities based on the volume flow rates measured with a flowmeter and examined measurement accuracy.

**Results:**

When the acrylic pipe was the size of the thoracicoabdominal or cervical artery and the ratio of pixel size for the pipe was set at 30% or lower, spatially-averaged velocity measurements were highly accurate. When the pixel size ratio was set at 10% or lower, maximum velocity could be measured with high accuracy. It was difficult to accurately measure maximum velocity of the 3-mm pipe, which was the size of an intracranial major artery, but the error for spatially-averaged velocity was 20% or less.

**Conclusions:**

Flow velocity measurement accuracy of 3D cine PC MR imaging for pipes with inner sizes equivalent to vessels in the cervical and thoracicoabdominal arteries is good. The flow velocity accuracy for the pipe with a 3-mm-diameter that is equivalent to major intracranial arteries is poor for maximum velocity, but it is relatively good for spatially-averaged velocity.

## Introduction

Magnetic resonance (MR) angiography can be used to visualize the shape of blood vessels and the hemodynamics in a non-invasive manner. Representative methods include the time of flight (TOF) method and the phase contrast (PC) method. The TOF method utilizes the phenomenon in which blood that flows into the imaging area presents a high intensity signal, and the PC method utilizes the phase shift of a spin that moves within the gradient magnetic field.^1^ The advantage of the former method is the high spatial resolution and relatively short acquisition time; thus, this method is widely used in clinical settings.^2–6^ On the other hand, the advantage of the latter method is that it is sensitive to slow flow and allows for quantification of the flow velocity.^7–9^ In addition to three dimensional (3D) imaging, PC MR angiography records velocity in three directions, allowing the calculation of velocity vectors.^10^ Furthermore, by imaging with a synchronized electrocardiogram, the hemodynamics associated with the cardiac cycle can be examined.^11^ This technology could be useful in predicting a future occurrence, in making early diagnoses, and in predicting the prognosis for vascular lesions.^12^

Phase images that are accompanied with velocity information can be obtained with PC MR angiography. To understand hemodynamics from these images, there are two methods. The first method calculates the velocity vector for each time phase of each pixel from 3D phase images. It is called magnetic resonance fluid dynamics (MRFD).^12–14^ The second method is a numerical analysis/simulation method called computational fluid dynamics (CFD).^15^ In this method, the shape of the target blood vessel is elucidated, and the appropriate volume flow rate (VFR) is applied to the inlet and outlet of this flow. The formula of the fluid movement is solved using a computer, making the flow observable. In either method, to obtain accurate hemodynamic information, high spatial resolution, temporal resolution, and signal intensity are required. These are not only contradictory to the imaging time, but they are also restricted by the technology of the MR scanner.

In the present study, we aimed to elucidate the effect of spatial resolution of 3D cine PC MR imaging on the accuracy of the blood flow analysis, and to clarify the optimal spatial resolution setting.

## Materials and Methods

### Flow phantom mimics the blood vessel diameter of humans

Flow phantom was prepared by using five types of acrylic pipes (inner diameters: 15, 12, 9, 6, and 3 mm) that mimic blood vessel diameters found in humans. These pipes were arranged so that they would pass through an acrylic container with a diameter of 20 cm and a depth of 10 cm. The container was filled with 1% agarose containing a gadolinium contrast agent (0.025 mol/L). The T_1_ relaxation time of the agarose with contrast agent was about 1,000 ms, while its T_2_ relaxation time was about 100 ms. To create a laminar flow, the sufficient entrance region was secured for the inlet of each pipe.

**Fig 1.**
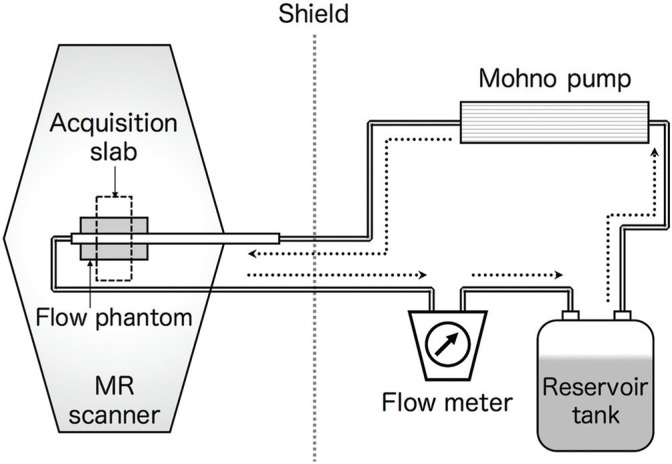
Schematics of the flow phantom. The dotted arrows indicate the flow direction. The fluid is initially removed from the reservoir tank by suction and then propelled by the Mohno pump; it flows through the acquisition slab in the magnetic resonance (MR) scanner, then through the flow meter, and, finally, it flows back to the tank.

We used an aqueous solution with 40% weight glycerine as the working fluid that passed through the acrylic pipe, and its temperature was adjusted to mimic the viscosity of human blood. A vibration viscometer (VM-10A; SEKONIC Corp., Tokyo, Japan) was used to measure viscosity, and in this experiment, the value was 3.57 mPa·s. A Mohno pump (NHL30; HEISHIN Ltd., Kobe, Japan) was used to circulate the fluid as a steady flow in the acrylic pipe (Fig. 1). The average flow velocity on the imaging section was set at 30 cm/s, and the VFR measurements were made with a Coriolis flow meter (FD-SF1 or FD-SF8; KEYENCE Corp., Tokyo, Japan) over time.

### MR imaging protocol

MR images were obtained using a 3-Tesla MR machine (MAGNETOM Verio 3T; Siemens healthcare Gmbh, Erlangen, Germany). The inner diameter of the acrylic pipe used as the imaging target determined how the receiving coil was set up. When imaging an acrylic pipe with inner diameters of 15 mm and 12 mm, a body matrix and a spine matrix coil were both used. When imaging an acrylic pipe with an inner diameter of 9 mm, a neck matrix coil was used. When imaging an acrylic pipe with inner diameters of 6 mm and 3 mm, a 12-ch Head matrix coil was used.

3D cine PC MR imaging was used a prototype sequence. Parameters for the 3D cine PC MR imaging were as follows: repetition time (TR) = minimum (29.16–39.80 ms); echo time (TE) = minimum (3.76–5.32 ms); flip angle = 15 degrees; bandwidth (BW) = 471–473 Hz/pixel; field of view (FOV) = 160 × 160 mm; number of slabs = 1; numbers of slices per slab = 12; slab orientation = axial, parallel acquisition technique (PAT) mode = GRAPPA; PAT factor = 2; number of segments = 1; cine phase number = 18–25; velocity encoding (VENC) = 80 cm/sec; VENC directions = 3 (foot-head [FH], right-left [RL], anterior-posterior [AP]); and acquisition time = 4:40–11:20 ms. Spatial resolution was changed by setting the matrix size at 80 × 80, 96 × 96, 128 × 128, 160 × 160, and 240 × 240. With these settings, slice thickness was set at 0.67, 1.00, 1.25, 1.67, and 2.00 mm, respectively. Therefore, voxel size became 0.67 × 0.67 × 0.67, 1.00 × 1.00 × 1.00, 1.25 × 1.25 × 1.25, 1.67 × 1.67 × 1.67, and 2.00 × 2.00 × 2.00 mm. A simulated electrocardiogram signal was generated in the MR scanner for 3D cine PC MR imaging. One heartbeat was set at 800 ms, and imaging was performed by the prospective gating method.

### Analytical methods

We performed MRFD using three-directional phase images obtained from the 3D cine PC MR imaging with a flow visualization and analysis software (Flova; Renaissance of Technology Corporation, Hamamatsu, Japan) as described below. We used magnitude images (rephrased images) obtained from 3D cine PC MR imaging for the segmentation of the pipe geometry. We created the pipe geometry based on the maximum intensity projection (MIP) images using the region growing method and the marching cube method. Each velocity was measured near the center of the acquired 12 slices. Spatially-averaged velocity was defined as average flow velocity inside of the acrylic pipe in one phase of the cardiac cycle, and the maximum velocity was defined as the largest value near the center of the acrylic pipe. In addition, both flow velocities were calculated as temporal average flow velocities over all time phases. These values were calculated through phase correction.^16^ By multiplying spatially-averaged velocity by the cross-sectional area, measurements can be converted to VFR. In addition, as maximum flow velocity presents the value of the largest pixel in the acrylic pipe, it can be an indicator to evaluate the accuracy of the digitized data.

The gold standard to calculate spatially-averaged velocities is attained by dividing the VFRs measured with the Coriolis flowmeter over time by the cross-sectional area of the acrylic pipe. Because this experiment maintained a steady laminar flow, we assumed it lead to Hagen-Poiseuille flow. Therefore, maximum velocity was assumed twice that of the average flow velocity from Hagen-Poiseuille equation.^17^ Spatial resolution was changed for each acrylic pipe, and spatially-averaged velocity and maximum velocity were obtained from results of MRFD analysis and compared with the standard. In addition, the following equation was used to calculate the error rate and accuracy of each measurement:

Error rate=(standard value−measured value/standard value)×100

### Statistical analysis

We employed the independent t-test for statistically significant difference to compare the flow velocity acquired with a 3D cine PC MR imaging and the actually measured flow velocity. A p value less than 0.05 was regarded as statistically significant.

## Results

**Fig 2.**
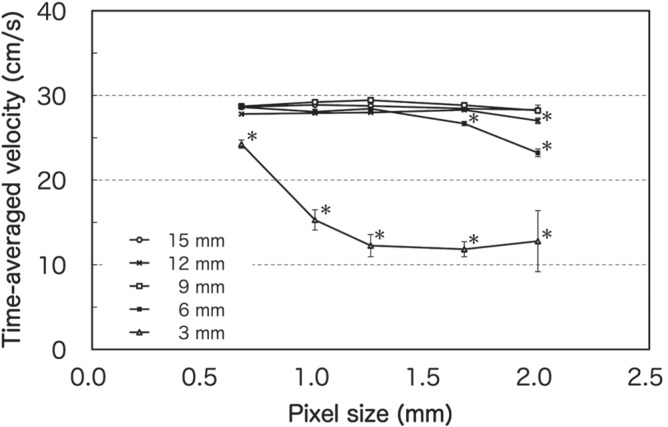
Results of spatially-averaged velocity averaged temporally obtained from three-dimensional cine phase contrast magnetic resonance (3D cine PC MR) imaging. Single asterisk (*P* value less than 0.05) indicates a statistical difference between the flow velocity acquired with a 3D cine PC MR imaging and the actually measured flow velocity.

**Fig 3.**
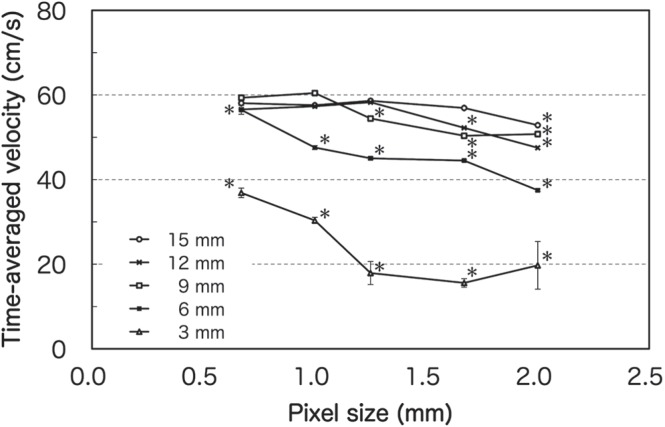
Results of maximum velocity averaged temporally obtained from three-dimensional cine phase contrast magnetic resonance (3D cine PC MR) imaging. Single asterisk (*P* value less than 0.05) indicates a statistical difference between the flow velocity acquired with a 3D cine PC MR imaging and the actually measured flow velocity.

**Fig 4.**
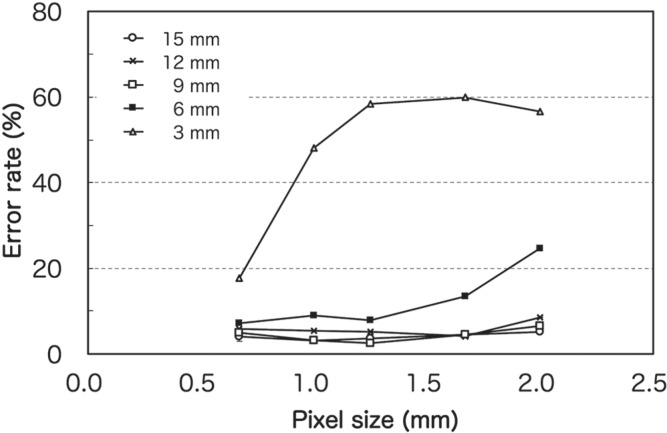
An error rate of spatially-averaged velocity averaged temporally obtained from three-dimensional cine phase contrast magnetic resonance (3D cine PC MR) imaging.

**Fig 5.**
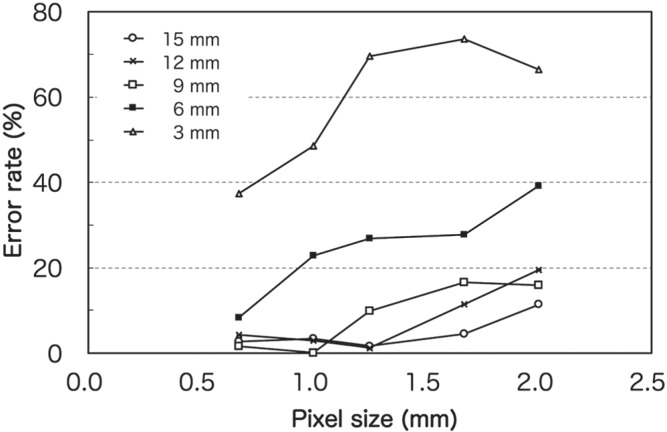
Error rate of maximum velocity averaged temporally obtained from three-dimensional cine phase contrast magnetic resonance (3D cine PC MR) imaging.

Temporally averaged spatially-averaged flow velocities and maximum flow velocities obtained from 3D cine PC MR imaging of the acrylic pipe with flowing fluid are shown in Figs. 2 and 3. The single asterisk in Figs. 2 and 3 (p value less than 0.05) indicates a statistical difference between the flow velocity acquired with a 3D cine PC MR imaging and the actually measured flow velocity. Each error rates were also shown in Figs. 4 and 5. The horizontal axis of the graph shows the pixel size (0.67, 1.00, 1.25, 1.67, and 2.00 mm), which was calculated by dividing FOV by matrix size. The vertical axis represents temporally averaged flow velocity (cm/s) for all time phases.

In the 15-mm-diameter acrylic pipe, spatially-averaged velocity was close to the designed average flow velocity of 30 cm/s for all pixel sizes, and the error rate was 5% or less. Maximum velocity presented a lower value when the pixel size was set at 2.00 mm, and the error rate was 10% or higher. With other pixel sizes, the maximum velocities became 60 cm/s, which was twice that of the average flow velocity.

In the 12-mm-diameter acrylic pipe, similar to the experimental results from the 15-mm-diameter acrylic pipe, spatially-averaged velocity was close to the designated mean flow velocity of 30 cm/s at all pixel sizes; however, the error rate was higher. Maximum velocity was low when using the pixel size of 1.67 mm or higher; when the pixel size was 2.00 mm, the velocity was 50 cm/s or lower. The error rate with the pixel size of 2.00 mm was about 20%.

In the 9-mm-diameter acrylic pipe, the spatially-averaged velocity was close to the average flow velocity of 30 cm/s for any pixel size. The error rate became 5% or higher when the pixel size was set at 2.00 mm, but was below 5% for all the other pixel sizes. The maximum velocity became low when pixel size was 1.25 mm or higher, and the error rate became 10% or higher.

In the 6-mm-diameter acrylic pipe, spatially-averaged velocity became low when the pixel size was 1.67 mm, and it became 25 cm/s or less at 2.00 mm. In addition, the error rate became 10% or higher when the pixel size became 1.67 mm, and it reached close to 25% with 2.00 mm. The maximum velocity was close to the standard value when the pixel size was 0.67 mm, but it was underestimated at all the other pixel sizes. The error rate increased with pixel size; when the pixel size became 2.00 mm, the error rate increased to ∼40%.

In the 3-mm-diameter acrylic pipe, spatially-averaged velocity did not approach the designated average flow velocity of 30 cm/s for any pixel size. When the pixel size was 0.67 mm, the error rate was 20% or less. With any other pixel size, the error rate increased to the 40–60% range. Analytical results of maximum velocity showed the same trend as the results of spatially-averaged velocity, and for any pixel size, the error rate was 35% or more.

## Discussion

There are many studies on 3D cine PC MR images using flow phantom, which mimics the diameter and shape of human blood vessels. To the best of our knowledge, this is the first report to evaluate the influence of spatial resolution on the measurement accuracy of blood flow analysis. The present study utilized acrylic pipes with different inner diameters that mimicked human blood vessel sizes. The resulting data elucidated the relationship between spatial resolution of 3D cine PC MR imaging and flow velocity measurement accuracy.

When the ratio of the pixel size to the inner diameter is set to less than 30%, we obtain the error rate of the spatially-averaged velocity as less than 10%. Similarly, when the ratio of the pixel size to the inner diameter is set to less than 10%, we obtain the error rate of the maximum velocity as less than 10%. This is almost in agreement with the conclusion derived from the results of a statistical test. The measurements are lower than the actual measurement value if the ratio of the pixel size to the inner diameter is greater than it. It was assumed that the measurements were influenced by the partial volume effect. The spatially-averaged velocity is calculated from the total number of pixels accounting for the cross section of the acrylic pipe. In contrast, the maximum velocity is calculated by a value in one pixel. Therefore, the partial volume effect greatly influences the measurement accuracy of the maximum velocity, and it is supposed to be the cause of the difference in the measurement values.^18^

The inner diameter of the common carotid artery is 6–8 mm, and, after branching, the internal and external carotid arteries are much thinner than the common carotid artery. If possible, it is desirable that the pixel size is set to be approximately 0.5 mm. The results obtained when using an acrylic pipe with an inner diameter of 3 mm were similar to those when the pixel size was 0.67 mm; in other words, when the ratio of pixel size to the inner diameter of acrylic pipe was set at ∼22%, the error rate of the spatially-averaged velocity was 20% or less and the error rate for the maximum flow velocity was ∼40%. A decrease in the pixel size might have lowered the signal to noise ratio and the measurement accuracy.

Machida et al. examined the relationship of PC cine MR imaging and the spatial factors by comparing the former with the intraluminal Doppler guidewire method.^19^ They generated constant flow velocities in a tube with a 4-mm diameter and examined a correlation of both measurement methods. The results showed that there is an excellent correlation between the PC cine-MRI and Doppler guidewire measurements of constant flow velocity. Moreover, the measurement accuracy of the spatially-averaged velocity and maximum velocity decreased sequentially as the spatial resolution decreased. These results show the same trend as our results.

We acquired three-dimensional MR images in our study. It is difficult to measure the hemodynamics of the complex vessel form, because two-dimensional MR imaging cannot acquire a high spatial resolution of the slice direction. For hemodynamic analysis of human blood vessels, isotropic voxel acquisition and 3D MR imaging might be necessary.

The results of the present experiment showed that MRFD analysis using 3D cine PC MR dataset can be used to accurately measure the hemodynamics of the thoracicoabdominal and neck arteries. Unfortunately, it remained difficult to accurately measure the intracranial arterial hemodynamics. However, spatially-averaged velocity was averaged flow velocities for the cross-sectional area of blood vessels; even with the ratio of pixel size against the blood vessel diameter being 30% or less, high measurement accuracy can be maintained. Therefore, when spatially-averaged velocities at the inlet and outlet are converted to VFR, CFD using these values as boundary conditions are estimated to provide us with accurate hemodynamics of intracranial arteries.

Naito et al. compared intracranial aneurysms using both “MRFD analysis” and “CFD analysis with high spatial resolution”.^20^ The results showed that the flow pattern and the wall shear stress (WSS) were relatively similar, but maximum WSS did not show a correlation. MRFD analysis can be used for intracranial aneurysms that have grown enough to be evaluated.

There were limitations in the present experiment. As we were mimicking human hemodynamics, pulsatile flow would have been preferred over the steady flow that was used. However, the objective of the present study was to clarify the effect of spatial resolution on the results of blood flow analysis; thus, the VFR of the fluid inside of the acrylic pipe was required. Consequently, we used a steady flow that could be accurately measured with a Coriolis flow meter.

## Conclusion

Results of fluid flow analyses for flow phantoms with varying diameters using 3D cine PC MR imaging from a 3-T MR imaging scanner showed that when the ratio of pixel size could be set at 30% or less, spatially-averaged velocity could be measured with high accuracy. A pixel size ratio of 10% or less allowed for accurate measurements of maximum velocity. These parameters fit the results from the pipes that were the equivalent size of the arteries in the neck and thoracicoabdominal regions. On the contrary, when performing fluid flow analysis for intracranial-sized pipes, accurate measurements of maximum velocity were difficult to obtain. However, the spatially-averaged velocity was obtained with high accuracy, so we would be able to use this information as a boundary condition of CFD analysis. By this CFD method, the hemodynamics of intracranial arteries could be more accurately analyzed than results of MRFD analysis.
